# Surgical outcome and prognostic factors in spinal cord ependymoma: a single-center, long-term follow-up study

**DOI:** 10.1177/17562864211055694

**Published:** 2021-11-10

**Authors:** Oliver Gembruch, Mehdi Chihi, Merle Haarmann, Ahmet Parlak, Marvin Darkwah Oppong, Laurèl Rauschenbach, Anna Michel, Ramazan Jabbarli, Yahya Ahmadipour, Ulrich Sure, Philipp Dammann, Neriman Özkan

**Affiliations:** Department of Neurosurgery, University Hospital Essen, University of Duisburg-Essen, Hufelandstrasse 55, 45122 Essen, Germany; Department of Neurosurgery, University Hospital Essen, University of Duisburg-Essen, Essen, Germany; Department of Neurosurgery, University Hospital Essen, University of Duisburg-Essen, Essen, Germany; Department of Neurosurgery, University Hospital Essen, University of Duisburg-Essen, Essen, Germany; Department of Neurosurgery, University Hospital Essen, University of Duisburg-Essen, Essen, Germany; Department of Neurosurgery, University Hospital Essen, University of Duisburg-Essen, Essen, Germany; Department of Neurosurgery, University Hospital Essen, University of Duisburg-Essen, Essen, Germany; Department of Neurosurgery, University Hospital Essen, University of Duisburg-Essen, Essen, Germany; Department of Neurosurgery, University Hospital Essen, University of Duisburg-Essen, Essen, Germany; Department of Neurosurgery, University Hospital Essen, University of Duisburg-Essen, Essen, Germany; Department of Neurosurgery, University Hospital Essen, University of Duisburg-Essen, Essen, Germany; Department of Neurosurgery, University Hospital Essen, University of Duisburg-Essen, Essen, Germany

**Keywords:** neurological outcome, outcome prediction, predictors, spinal ependymoma, surgery

## Abstract

**Objective:**

Spinal cord ependymomas account for 3–6% of all central nervous system tumors and around 60% of all intramedullary tumors. The aim of this study was to analyze the neurological outcome after surgery and to determine prognostic factors for functional outcome.

**Patients and Methods:**

Patients treated surgically due to a spinal cord ependymoma between 1990 and 2018 were retrospectively included. Demographics, neurological symptoms, radiological parameters, histopathology, and neurological outcome (using McCormick Score [MCS]) were analyzed. Possible prognostic factors for neurological outcome were evaluated.

**Results:**

In total, 148 patients were included (76 males, 51.4%). The mean age was 46.7 ± 15.3 years. The median follow-up period was 6.8 ± 5.4 years. The prevalence was mostly in the lumbar spine (45.9%), followed by the thoracic spine (28.4%) and cervical spine (25.7%). Gross-total resection was achieved in 129 patients (87.2%). The recurrence rate was 8.1% and depended on the extent of tumor resection (*p* = 0.001). Postoperative temporary neurological deterioration was observed in 63.2% of patients with ependymomas of the cervical spine, 50.0% of patients with ependymomas of the thoracic spine, and 7.4% of patients with ependymomas of the lumbosacral region. MCS 1–2 was detected in nearly two-thirds of patients with cervical and thoracic spinal cord ependymoma 36 months after surgery. Neurological recovery was superior in thoracic spine ependymomas compared with cervical spine ependymomas. Poor preoperative functional condition (MCS >2), cervical and thoracic spine location, and tumor extension >2 vertebrae were independent predictors of poor neurological outcome.

**Conclusion:**

Neurological deterioration was seen in the majority of cervical and thoracic spine ependymomas. Postoperative improvement was less in thoracic cervical spine ependymomas compared with thoracic spine ependymomas. Poor preoperative status and especially tumor extension >2 vertebrae are predictors of poor neurological outcome (MCS >2).

## Introduction

Spinal cord ependymomas are usually slow-growing tumors arising from ependymal cells of the central canal of the spinal cord.^1^ Ependymomas account for 3–6% of all central nervous system tumors and are the most frequent spinal cord neoplasm in adults, presenting 60% of all intramedullary tumors.^2–5^

Symptom presentation is related to the tumor location and can include radicular or local pain, motor weakness of the extremities, hypoesthesia, gait disturbance, and sphincter or sexual dysfunction.^6–8^ Non-specificity of symptoms can lead to adaptation to symptoms and late diagnosis. Cervical tumors can present symptoms of upper or lower extremities, due to the corticospinal tract or dorsal column being affected.^9^ Symptom duration depends on tumor location and symptom characteristics, with back pain being the most common symptom. The average symptom duration described in the literature is around 2 years.^10–12^ In rare cases, an acute deterioration of the symptoms can be provoked by intratumoral hemorrhage.^13–15^

The World Health Organization (WHO) grading system includes three ependymoma subtypes: WHO grade I: the myxopapillary ependymoma and the subependymoma; WHO grade II: ‘classic’ ependymoma including papillary, clear cell, and tanycytic subtypes; and WHO grade III: anaplastic ependymoma.^16^

The ‘classic’ ependymoma is the most common in the spinal cord with a frequency of 55–75%.^9,13^ Benign ependymomas (WHO grade I) and semi-benign ependymomas (WHO grade II) have well-defined margins that allow microsurgical tumor removal without damaging the spinal cord tissue. In contrast, anaplastic ependymomas (WHO III) are infiltrative and only subtotal resection is possible.^13,17,18^ The prognosis based on WHO grading alone is difficult due to the heterogeneity of ependymomas and its tumor characteristics.^1,6,13^

The long-term survival and prognostic factors for tumor-free survival in spinal cord ependymomas have been thoroughly investigated.

According to current gold standard, surgical resection remains the therapy of choice in spinal cord ependymomas, especially for patients presenting with neurological impairments.

Postoperative neurological deterioration remains a major problem that might be reduced further as surgical techniques continue to advance.^19^ Despite the well-known neurological deterioration after surgery, studies focusing on predictors of neurological outcome are scarce because of the small sample size.^6,9,20,21^

This study is one of the largest single-center studies of a European neurosurgical center and aims to describe the tumor entity and the surgical course. In addition, we focused on factors causing postoperative functional deterioration and tried to identify predictive factors with impact on the postoperative neurological outcome.

## Patients and methods

### Study population

A retrospective analysis of the electronic database ‘spinal neoplasm’ evaluating the clinical and radiological data and operative reports of patients suffering from a spinal ependymoma who attended to our department between 1990 and 2018 was performed. Only patients suffering from primary spinal cord ependymoma were included in the analysis. Patients with primary cerebral ependymoma and secondary spinal cord metastasis or leptomeningeal metastasis were excluded.

### Evaluated parameters

The demographics, symptom duration until surgery, neurological symptoms such as pain, sensory deficits, motor deficits (monoparesis/hemiparesis and paraparesis), gait disturbance, and bladder dysfunction for each case were noted. Radiological parameters including tumor location (intramedullary and extramedullary, cervical, thoracic, and lumbar), tumor size in terms of size expanding over the number of vertebrae, and volume (cm^3^) according to preoperative magnetic resonance imaging (MRI) were evaluated. Gross-total resection (GTR) was defined as complete tumor removal, showing no tumor remnants in the early postoperative MRI with contrast. Subtotal tumor resection (STR) was present if the early postoperative MRI showed tumor remnants.

Patients’ neurological status was evaluated using the McCormick Score (MCS I: neurologically normal; mild focal deficit not significantly affecting the function of the involved limb; mild spasticity or reflex abnormality; normal gait; MCS II: presence of sensorimotor deficit affecting the function of the involved limb; mild to moderate gait difficulty; severe pain or dysesthetic syndrome impairing patient’s quality of life; still functions and ambulates independently; MCS III: more severe neurological deficit; requires cane/brace for ambulation or significant bilateral upper extremity impairment; may or may not function independently; MCS IV: severe deficit; requires wheelchair or cane/brace with bilateral upper extremity impairment; usually not independent).^22^ The MCS was retrospectively derived from clinical data at the beginning of the observational study. Later, MCS was routinely used in clinical practice. The MCS was modified according to the current literature^20,23^ to allow a more valuable discrimination in postoperative neurological outcome: ‘good’ was defined as MCS I + II and ‘poor’ was defined as MCS III + IV. A cut-off value of MCS >2 was chosen because patients suffer from moderate neurological deficits with limitations in function. Furthermore, external aid may be needed. Patients with MCS ⩽2 do not show severe neurological deficits and do not need external help.

According to our clinical standard of care, neurological examination was performed routinely preoperatively, postoperatively on the last day at the hospital, and 3 months, 6 months, 12 months, 24 months, and 36 months after surgery. Furthermore, postoperative status including MCS was assessed 3, 5, and 10 years after surgery. Postoperative MRI was performed within 72 h after surgery. Further MRI controls were performed every 3–6 months.

### Surgical treatment

**Figure 1.**
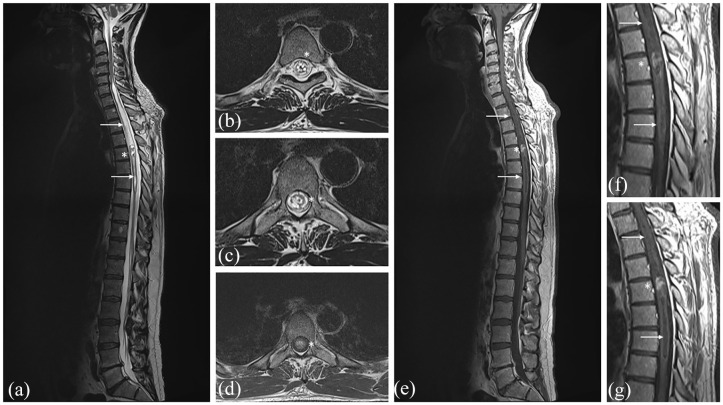
Preoperative T2-weighted MRI showing the intramedullary ependymoma (*) at Th 5 with intertumoral hemorrhage and edema of the spinal cord (→) from C7-Th7 (a-c). T1-weighted MRI with contrast showing the contrast enhancement of the ependymoma (d–g).

**Figure 2.**
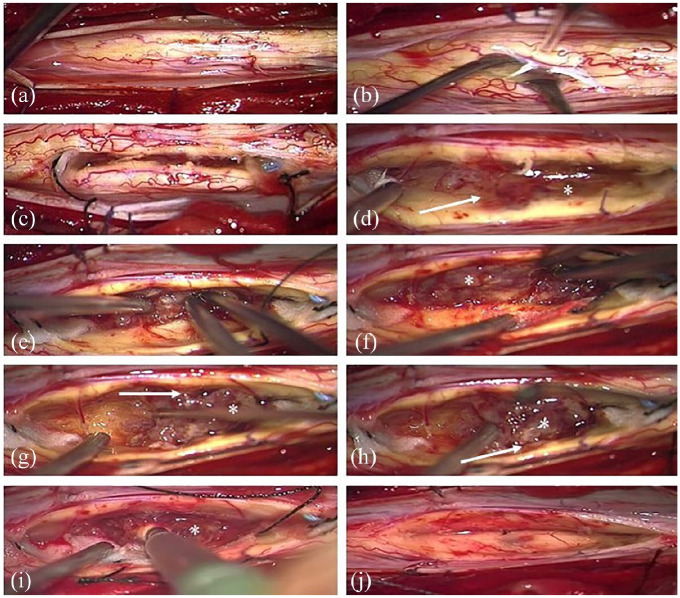
The spinal cord is exposed after dura opening and bulged due to the intramedullary tumor (a). Myelotomy performed medially (b). The cranial and caudal boundary (see tidal flats) of the tumor is prepared (c). The margins (→) of the grayish tumor (*) are well defined (d). Debulking of the tumor and piecemeal removal using a CUSA with preservation of the surrounding spinal cord tissue (e–i). Spinal cord after complete tumor removal (j).

**Figure 3.**
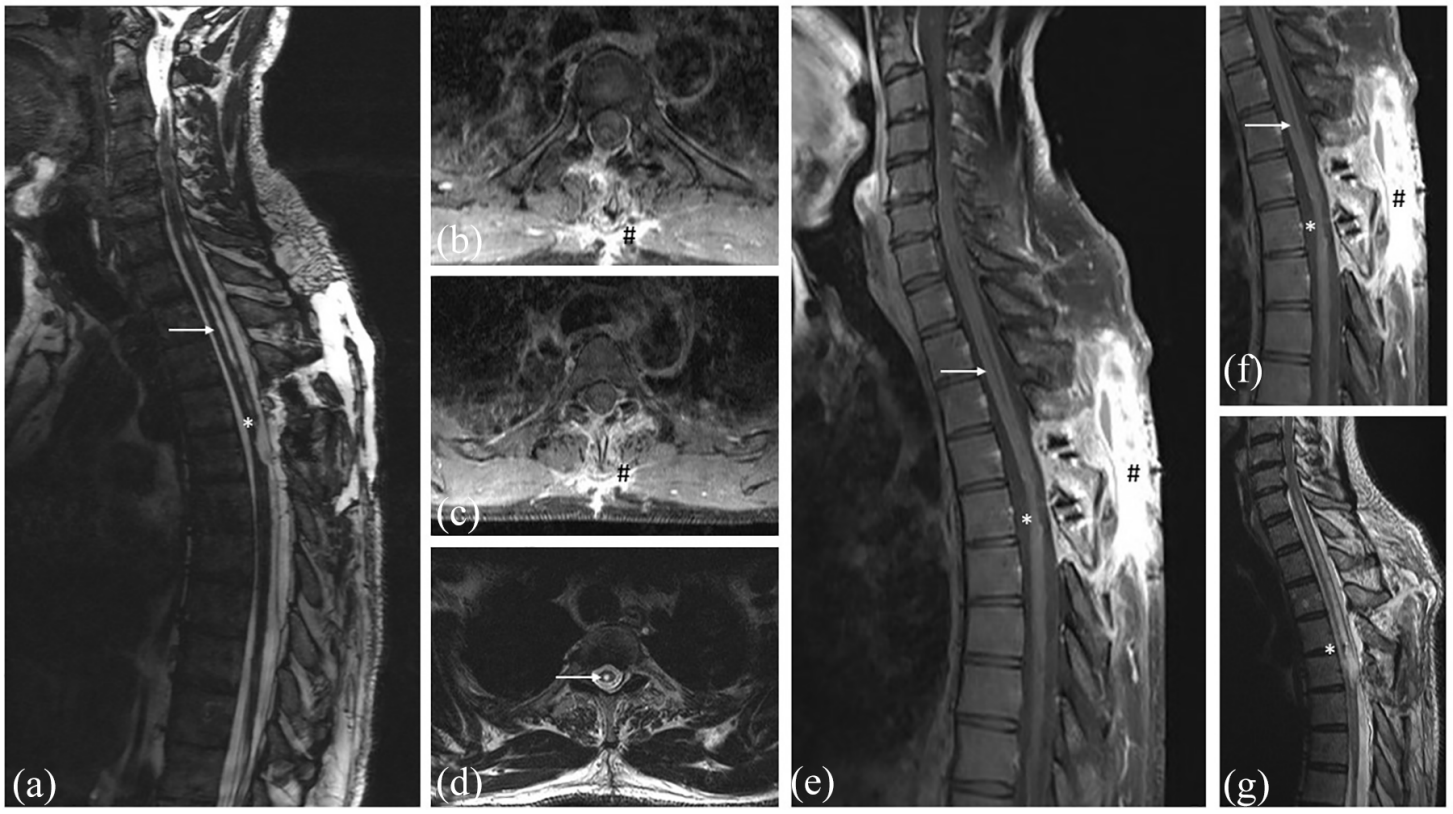
Early postoperative T2-weighted MRI without contrast (a + d) and T1-weighted MRI with contrast (b + c) 24 h after surgery with completely removed ependymoma. T1-weighted MRI with contrast (e + f) 6 months after surgery showing no tumor recurrence (*) despite the normal contrast enhancement at the dorsal approach (#). T2-weighted MRI without contrast showing the postoperative changes of the spinal cord (*) and the smaller edema of the spinal cord (→) (g).

The surgery was performed in microsurgical fashion using a standard dorsal approach in prone position for lesions located at the thoracal and lumbar spine. Semi-sitting position was favored in cervical spine tumors. Laminoplasty was routinely performed, whereas laminectomy was performed in cases where refixation of the laminae was deemed unfavorable (distinct osteoporosis or vertebral deformity). Hemilaminectomy was indicated in lateral located tumors and usually in lumbar filum terminal ependymomas. Surgical removal of the ependymoma was performed with the aid of intraoperative monitoring (somatosensory evoked potentials and motor evoked potentials). The tumor was removed piecemeal-like, beginning from the center to the well-defined margins and the surrounding spinal cord tissue (Figures 1–3). Patients were mobilized after a bed rest of 3 days to avoid postoperative cerebrospinal fluid fistula. Tumor analysis was performed at the Department of Neuropathology of the University Hospital Essen.

### Statistics

Data were analyzed using SPSS 25.0 (Statistical Package for the Social Sciences, SPSS Inc., Chicago, IL, USA) for Windows. Metric data were described by mean and standard deviation and nominal data by frequency and valid percentage. *P* values <0.05 in two-sided testing were considered significant.

Demographic, clinical, and radiographic parameters were analyzed in a univariate way regarding their association or correlation with preoperative and postoperative McCormick Score. Therefore, Pearson’s χ^2^ statistics or Fisher’s exact test was used for dichotomous variables. For non-normally distributed data, the Kendall’s tau-b was assessed for continuous and ordinal, Spearman’s rho for continuous and dichotomous, and Mann–Whitney *U* test for ordinal and continuous variables. Significant parameters selected through univariate analysis and parameters with *p* values <0.1 were subsequently evaluated using multivariate analysis.

The neurological outcome was analyzed based on the tumor location: cervical, thoracic, and lumbar. Patients who were lost to follow-up were not included in statistical analysis at those time points.

## Results

### Clinical characteristics

Over a period of 28 years, 148 patients [72 females (48.6%) and 76 males (51.4%)] suffering from spinal cord ependymoma underwent surgery in our institute. The mean age was 46.7 ± 15.3 years, ranging from 9 to 83 years. Four patients were 16 years and younger. The mean follow-up was 6.8 ± 5.4 years (up to 27 years). However, 13 patients (8.8%) were lost to follow-up 12 months after surgery and 23.0% (34 patients) 36 months after surgery.

The most frequently involved localization was the lumbar-sacral region (45.9%), followed by the thoracic (28.4%) and cervical region (25.7%). The tumor was located intramedullary in 59.5% (Table 1).

**Table 1.** Demographic, surgical, and tumor characteristics.

Patients’ characteristic		*P* value
Number of patients	148	
Age (years)	46.7 ± 15.3	
Sex (female)	72 (48.6%)	
Duration of symptoms (months)	29.4 ± 57.3	
Tumor characteristic		*P* value
Tumor location		
Intramedullary	88 (59.5%)	
Extramedullary	60 (40.5%)	
Tumor location		
Cervical	38 (25.7%)	
Thoracic	42 (28.4%)	
Lumbar	68 (45.9%)	
WHO grade I	70 (47.3%)	
WHO grade II	74 (50.0%)	
WHO grade III	4 (2.7%)	
Cervical: WHO grade (I/II/III)	(7/30/1)/38	*p* = 0.0001
Thoracic: WHO grade (I/II/III)	(17/24/1)/42	
Lumbar: WHO grade (I/II/III)	(46/20/2)/68	
Tumor recurrence		*P* value
Total tumor recurrence	12 (8.1%)	*p* = 0.024
Cervical spine	0/38 (0%)	
Thoracic spine	7/42 (16.7%)	
Lumbar spine	5/68 (7.4%)	
Tumor recurrence after GTR	5/129 (6.0%)	*p* = 0.0001
Tumor recurrence after STR	7/19 (36.8%)	
Surgical characteristics		*P* value
Surgical approach		
Laminoplasty	76 (51.4%)	
Laminectomy	70 (47.3%)	
Hemilaminectomy	2 (1.4%)	
GTR	129/148 (87.2%)	
Cervical Spine	33/38 (86.8%)	*p* = 0.100
Thoracic Spine	33/42 (78.6%)	
Lumbar Spine	63/68 (92.6%)	

GTR, gross-total resection; STR, subtotal tumor resection; WHO, World Health Organization.

**Graph 1.**
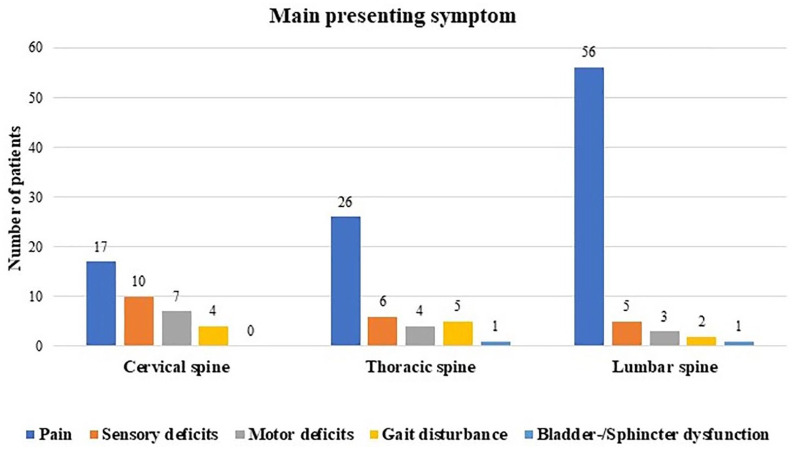
Main presenting symptoms according to tumor location.

The average duration of symptoms until surgical treatment was 29.4 ± 57.3 months. The presenting symptom was pain in 67.6%. Radiating pain was presented in the majority of the cases (53.5%), while back pain was described in 34.3%. In 12.1%, the symptom could not be specified by the patients. Sensory deficits and motor deficits were described in 14.2% and 9.5%. Gait disturbances were complained in 7.4%. Only two patients (1.4%) reported sphincter and bladder dysfunction. The main presenting symptom depended on tumor location. Pain was more often seen in patients with ependymomas located in the lumbar spine (83.8%). Sensory deficits (26.3%) and motor deficits (18.4%) are more common in cervical spine ependymomas. Gait disturbance was detected in cervical and thoracic spine ependymomas, whereas sphincter and bladder dysfunction were observed in thoracic and lumbar spine tumors (Graph 1).

**Graph 2.**
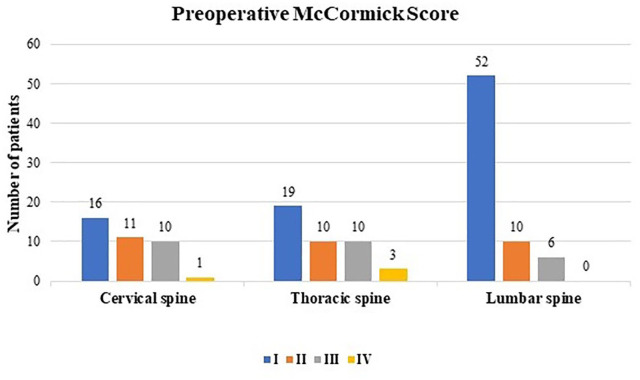
Preoperative McCormick Score (I–IV) in relation to the affected spine level.

The preoperative functional status according to MCS was generally good (MCS I: 58.8% and MCS II: 20.9%). Only a few patients presented with severe neurological impairments (MCS III: 17.6% and MCS grade IV: 2.7%) prior to surgery. Worse preoperative MCS (III + IV) was seen more often in patients with cervical and thoracic ependymomas (Graph 2).

### Surgery

Laminectomy was chosen in 47.3% of the cases and mainly performed in the 1990s and the early 2000s. Laminoplasty was the preferred approach in the later phase of the observation period.^24^ The GTR was achieved in 129 of cases (87.2%), and the STR was performed in 19 cases (12.8%). Lumbar spine ependymomas were most commonly resected via GTR (92.6%) followed by cervical spine ependymomas (86.8%) and thoracic spine ependymomas (78.6%) (Table 1).

### Surgical complications

A cerebrospinal fluid fistula prolonged the wound healing in 9 (6.1%) of 148 patients. There was no relation to the operative approach. Laminectomy was used in five cases and laminoplasty in four cases. The tumor was located in the cervical spine in two cases, in the thoracic spine in one case, in the thoracic-lumbar region in three cases, and in the lumbar spine in three cases. However, none of these patients required surgery and the fistula healed out completely with conservative treatment.

### Histopathology

Ependymomas (WHO grade I) were diagnosed in 47.3% and ependymomas (WHO grade II) in 50.0%. Anaplastic ependymoma was detected in 2.7%. Ependymomas (WHO grade I) were more commonly located in the lumbar region, whereas tumors (WHO grade II) were more often diagnosed in cervical spine (Table 1).

### Recurrence

Tumor recurrence was detected in 12 patients (8.1%) after a mean follow-up of 21.8 months (range, 1 month–4.6 years). One patient developed a spinal ependymoma 20 years after surgery at a completely different spinal location. A tumor recurrence occurred in 3.9% (5/129 cases) after GTR and in 36.8% after STR (7/119 cases), showing significantly higher rates of recurrence than after GTR (*p* = 0.0001). Histological examination confirmed a benign tumor (WHO grade I) in five cases, a semi-benign tumor (WHO grade II) in two cases and anaplastic ependymoma (WHO grade III) in five patients. All patients with recurrent tumor, except one, who were treated using radiotherapy after biopsy underwent a second surgery (Table 1).

### Adjuvant therapy

Each case was discussed at the interdisciplinary tumor board. Postoperative radiotherapy was routinely offered to all patients with ependymomas WHO grades II and III. Of these patients, 15.6% of the patients with a grade II ependymoma (10/64 patients) and 100% of the patients with a grade III ependymoma underwent postoperative radiotherapy (Table 1).

### Neurological outcome according to the spine level

#### Cervical spine

**Graph 3.**
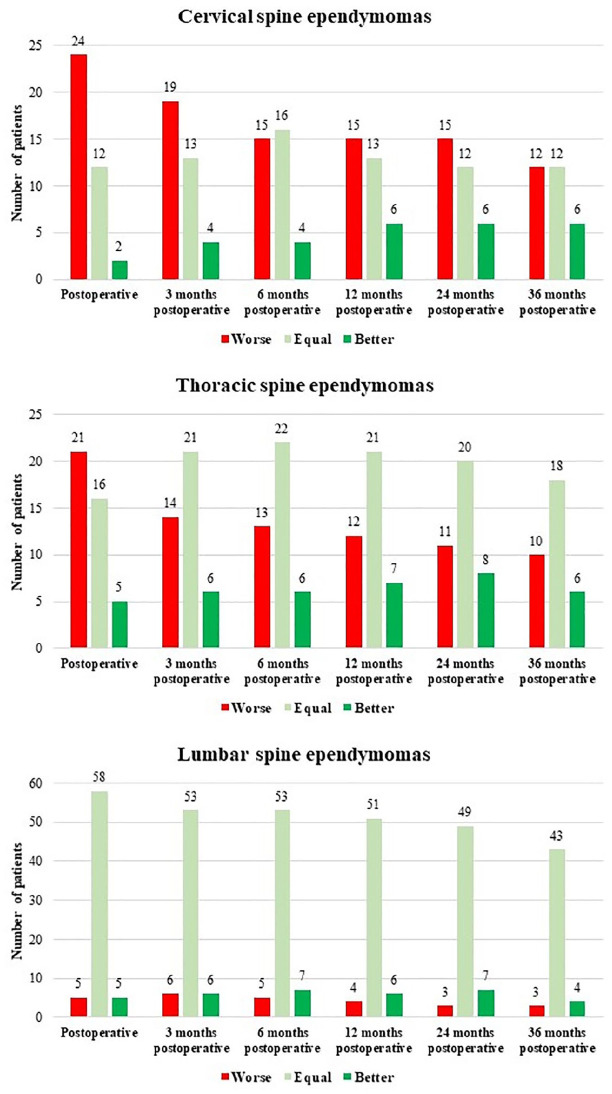
Postoperative neurological status according to different spine levels.

Preoperative MCS was ‘good’ in 71.1% and ‘poor’ in 28.91% (Table 2). Postoperative neurological deterioration was seen in the majority of the patients (63.2%). Of these, 40% did not reach preoperative neurological status (Graph 3). However, almost all patients (63.3%) recovered to the previous ‘good’ preoperative status. The ‘poor’ MCS consecutively decreased from 63.2% postoperatively to 48.6% 6 months and to 36.7% 36 months after surgery (Table 2).

**Table 2.** Postoperative functional outcome according to the MCS.

	MCS	Preoperative *n* = 148	Postoperative *n* = 148	3 months postoperatively *n* = 142	6 months postoperatively *n* = 141	12 months postoperatively *n* = 135	24 months postoperatively *n* = 131	36 months postoperatively *n* = 114
Cervical	MCS I + II	27 (71.1%)	14 (36.8%)	17 (47.2%)	18 (51.4%)	21 (61.7%)	20 (60.6%)	19 (63.3%)
	MCS III + IV	11 (28.9%)	24 (63.2%)	19 (52.8%)	17 (48.6%)	13 (38.3%)	13 (39.4%)	11 (36.7%)
Thoracic	MCS I + II	29 (69.0%)	23 (54.7%)	24 (58.5%)	24 (58.5%)	25 (62.5%)	24 (61.5%)	21 (61.8%)
	MCS III + IV	13 (31.0%)	19 (45.3%)	17 (41.5%)	17 (41.5%)	15 (37.5%)	15 (38.5%)	13 (38.2%)
Lumbar	MCS I + II	62 (91.2%)	63 (92.6%)	60 (92.3%)	60 (92.3%)	57 (93.4%)	55 (93.2%)	46 (92.0%)
	MCS III + IV	6 (8.8%)	5 (7.4%)	5 (7.7%)	5 (7.7%)	4 (6.6%)	4 (6.8%)	4 (8.0%)

MCS, McCormick Score.

#### Thoracic spine

Patients with thoracic spine ependymoma presented with ‘good’ preoperative MCS in 69.0%. The ‘good’ MCS decreased to 54.7% postoperatively and increased to 61.8% 36 months after surgery (Table 2). Neurological deterioration was seen in 50.0% postoperatively, while 38.1% remained stable and 11.9% reported direct postoperative improvement in neurological deficits. However, 36 months after surgery neurological deterioration was still present in 29.4%. Preoperative status was reached in 53.0%, while neurological improvement compared with the preoperative status was detected in 17.6% (Graph 3).

#### Lumbar spine

In contrast to patients suffering from cervical spine and thoracic spine ependymomas, preoperative MCS was ‘good’ in general (91.29%). A ‘poor’ preoperative MCS was only detected in 8.8%. Postoperative MCS remained ‘good’ in the majority of the cases (92.6%). A ‘good’ MCS was seen in 92.0% 36 months after surgery (Table 2).

Postoperative deterioration was only seen in 7.4%, while 85.2% showed stable neurological status or improvement in status after surgery (7.4%). Neurological deterioration was observed in only 6% 36 months after surgery, while 86.0% remained unchanged or improved their neurological status (8.0%) (Graph 3).

### Analysis of possible predictors of neurological outcome

#### Complete cohort

Univariate analysis of the total cohort revealed that poor preoperative functional condition (MCS > 2), WHO grades II and III, tumor volume (cm^3^), spine segment (cervical and thoracic spine), tumor extension >2 vertebras, and STR had a high significant impact on poor neurological outcome (*p* < 0.05).

In addition, neurological symptoms (pain, paresis, and ataxia) at onset of the disease were negatively associated with postoperative outcome (*p* < 0.05), whereas sensory disorders had no effect (*p* > 0.05) on patients’ outcome. Symptom duration also showed no significant correlation with poor neurological outcome (*p* > 0.05) (Supplementary Table 3).

Multivariate analysis confirmed that preoperative status (MSC >2) was a negative predictor of neurological outcome at all analyzed time points. Tumor extension >2 vertebrae was a negative predictor until 24 months after surgery. In addition, STR was also a negative predictor 36 months after surgery (*p* < 0.05) (Supplementary Table 3).

#### Cervical and thoracal spine ependymomas

The univariate analysis demonstrated that preoperative MCS >2 and tumor extension (>2 vertebrae) had significant association with poor neurological outcome at every evaluated time points (Supplementary Table 4).

The multivariate analysis showed an important impact of tumor extension >2 vertebrae on a poor functional outcome (postoperatively: 24 months after surgery (Supplementary Table 4).

#### Lumbar ependymomas

The univariate analysis of the lumbar ependymomas revealed preoperative MCS >2, pain, tumor extension (>2 vertebrae), and ataxia as potential predictive factors of poor neurological outcome (*p* < 0.05) (Supplementary Table 5).

Multivariate analysis revealed that preoperative MCS was a predictive factor postoperatively (*p* < 0.05). However, quality of this subgroup was limited due to the low number of evaluated patients and lost to follow-up 24 and 36 months after surgery (Supplementary Table 5).

## Discussion

The surgical treatment of spinal ependymomas remains challenging, as postoperative neurological deterioration plays a key role in prognosis. Up to now, several authors presented their surgical experience and their recommendation for surgical tumor removal or mass reduction.^2,4,7,25,26^

The known postoperative neurological deterioration of the majority of patients makes it necessary to define possible prognostic factors for functional outcome. Therefore, we tried to present our single-center experience on the surgical treatment of spinal cord ependymomas and evaluated possible prognostic factors for neurological deterioration. This single cohort outnumbers cohorts published in literature.

A mild predominance of males (52.4%) was detected in our study. The frequency of males ranged from 62% to 82% in other series.^20,22,25^ Ependymomas were mostly located in the lumbar region (45.9%). Lumbar ependymomas were described in 2.9–29.1% of the cases in the literature.^27,28^

Symptoms caused by the spinal ependymoma are unspecific. The most common symptom was pain in 67.6% of the cases, followed by sensory deficits in 14.2% and motor weakness in 9.5%. These results are similar to previously published reports.^6,18,29^ Symptom duration until surgery was, on average, 29.4 months. Late diagnosis is caused by the non-specificity of symptoms.^29,30^ Furthermore, authors described a misinterpretation of symptoms such as back pain or slow deterioration of other neurological symptoms with resulting lack of differential diagnosis, including a slow-growing spinal tumor.^31^ In addition, patients can adopt the slow worsening of neurological deficits at the early stage or comorbidities can cover these symptoms. Nevertheless, at the time of diagnosis, a considerable number of patients showed severe neurological deficits (MCS III = 17.6%) or were not able to walk (MCS IV = 2.7%). Boström *et al.*^18^ reported about 2% and Klekamp^6^ reported about 11.2% of patients who were unable to walk preoperatively. Li *et al.*^25^ reported about 26.2% of patients with MCS III and 6.2% of patients with MCS IV in a series of 210 patients suffering from a spinal cord ependymoma.

### Surgical treatment and tumor recurrence

In our series, complete resection of spinal cord ependymomas was seen in the early postoperative MRI in 87.2%, showing comparable results to the literature.^2,5,20,25,32^ However, GTR was most commonly achieved after tumor removal within the lumbar spine followed by the cervical spine. Rate of GTR was lowest in thoracic spine ependymomas. This might be caused by the anatomical differences between the spine segments. In the current literature, GTR, encapsulated tumors, and postoperative radiotherapy are reported as the most important prognostic factors for progression-free survival.^2,5–7,15,29,33^ Tumor recurrence was detected in 8.1% of the patients after a median of 21.8 months, leading to consecutive impairment of the spinal cord. This was also reported by Samii and Klekamp, who described higher rates of neurological deterioration in STR. The regrowth of tumor remnants was proposed as a possible explanation.^34^ However, the only significant predictor of recurrence-free survival is the degree of resection.^18^ In addition, it has been well established that an early start of adjuvant treatment after non-total resection prolongs progression-free survival.^35^

### Functional outcome

Change in surgical approach (laminectomy at the beginning of the period *versus* laminoplasty^24^ from the early 2000s onward) during the treatment period had no impact on neurological outcome.^5^ Neurological deterioration after surgery was most common in cervical and thoracic spine ependymomas due to the anatomical differences between the lumbar spine. Neurological deterioration after surgery was present in 63.2% of the cervical spine ependymomas and in 50.0% of the thoracic spine ependymomas compared with a worsening of 7.4% of the lumbar spine ependymomas. However, direct postoperative improvement of the neurological function was detected in 5.2%, 11.9%, and 7.4%, respectively. Neurological improvement after rehabilitation 36 months after surgery was seen especially in thoracic spine ependymomas. Of those, 53% reached the preoperative status, while 17.6% were better than preoperative status. Thoracic spine ependymomas showed poor MCS after 36 months in 61.8%. Some authors propose that the anatomy of the spinal cord (smaller volume of the thoracic spinal cord, compared with the cervical spinal cord) is reasonable for the worse recovery.^6^ However, neurological improvement might be also influenced by tumor recurrence at the time of follow-up. In our analysis, tumor recurrence was observed after 21.8 months. Nevertheless, good MCS was observed in the majority of lumbar spine ependymomas and in 63.3% of the cervical and 61.8% of the thoracic spine ependymomas 36 months after surgery. Cervical spine ependymomas presented less improvement than thoracic spine ependymomas. This is comparable with the findings of Klekamp,^6^ who described neurological deterioration of 67.5% of the patients with intramedullary ependymomas and of 16.6% of the patients with filum terminale ependymomas.^27^ Transient neurological deterioration was evaluated in 40% and 8.3%, respectively.^6,27^ Similar to our study, Klekamp^6^ found a higher rate of postoperative permanent morbidity in patients with tumors of thoracic spine.

### Predictors of poor neurological outcome

However, there are some factors with potential risk for permanent neurological deficits. In our series as well as in others, the preoperative neurological status bears an increased risk of poor neurological outcome.^6,7,18,20,36,37^ Preoperative neurological deficit reflects the damage of the spinal cord caused by the tumor, and the operative procedure increases this damage. In addition, regeneration of the neurological function is limited, especially if the preoperative poor neurological status persists over a longer time. Early diagnosis and referral to specialized surgical centers might also improve the postoperative outcome. Epstein *et al.* also call for early surgery based on their findings.

Another predictive factor for poor neurological outcome until follow-up of 24 months is tumor extension >2 vertebrae. The extent of tumor resection and the consecutive injury of the spinal cord may be reasonable. Prokopienko *et al.*^20^ showed in their study that neurological outcome is worse in tumors extending over three spinal levels. Wang *et al.*^38^ evaluated that tumor size is a predictive factor for worse neurological outcome if the tumor is larger than 4 cm.

Tumor location was also a predictor of poor neurological outcome. Tumors located in the cervical and thoracic spine were less likely to achieve good neurological outcome compared with lumbar spine ependymomas. These findings are similar to the current literature.^39^ For example, Samuel *et al.*^19^ showed similar results analyzing treatment of 63 patients with intramedullary spinal cord tumors. Interestingly, Wostrack *et al.*^21^ evaluated that cervically located ependymomas causing transient deficits were more frequent but failed to demonstrate that cervical tumor location is a predictor of permanent neurological deficits.

Finally, STR showed a tendency to be a predictor of neurological outcome 12 and 24 months after surgery. It was detected as a negative predictor of neurological outcome 36 months after surgery. The majority of tumor recurrence occurred in patients with STR after a mean follow-up of 21.8 months. In those cases, second surgery was offered to the patients and a second neurological deterioration was detected.

### Study limitations

Various limitations must be addressed. First, this is a retrospective, non-randomized study with its associated inherent bias. Second, data were analyzed from our retrospective electronic database ‘spinal neoplasm’, in which the patient’s electronic data, surgical reports, and radiological data were collected. Nevertheless, incomplete data bear an additional limitation and risk of selection bias. Univariant and multivariant analysis was not useful due to the ongoing lost to follow-up 36 months after surgery, caused by the retrospective character of the study. In addition, the study encompassed a long epoch in time, in which patients were treated by different neurosurgeons creating another confounding factor. Furthermore, some surgical techniques have changed over these years in terms of minimally invasive approaches or the use of intraoperative electrophysiology. In addition, lower image quality of MRI during the beginning of the observational study has to be acknowledged and might have influenced the quality of the data.

## Conclusion

Spinal cord ependymomas present different clinical features according to their location. The surgical treatment of these tumors is associated with a considerable risk of postoperative neurological deterioration, which is most common in cervical and thoracal spine ependymomas. However, postoperative improvement is likely in half of these patients.

The preoperative neurological status, tumor location at the cervical spine, and STR are negative predictors of the postoperative MCS. Therefore, the surgical treatment of spinal cord ependymomas before further neurological deterioration is recommended.

## Supplemental Material

sj-docx-1-tan-10.1177_17562864211055694 – Supplemental material for Surgical outcome and prognostic factors in spinal cord ependymoma: a single-center, long-term follow-up study Click here for additional data file. Supplemental material, sj-docx-1-tan-10.1177_17562864211055694 for Surgical outcome and prognostic factors in spinal cord ependymoma: a single-center, long-term follow-up study by Oliver Gembruch, Mehdi Chihi, Merle Haarmann, Ahmet Parlak, Marvin Darkwah Oppong, Laurèl Rauschenbach, Anna Michel, Ramazan Jabbarli, Yahya Ahmadipour, Ulrich Sure, Philipp Dammann and Neriman Özkan in Therapeutic Advances in Neurological Disorders
