# Supplementary material for: Surgical outcome and prognostic factors in spinal cord ependymoma: a single-center, long-term follow-up study
Source: Ther Adv Neurol Disord. 2021 Nov 10;14:17562864211055694. doi: 10.1177/17562864211055694 (PMC8591778; doi:10.1177/17562864211055694)
Supplement: sj-docx-1-tan-10.1177_17562864211055694 – Supplemental material for Surgical outcome and prognostic factors in spinal cord ependymoma: a single-center, long-term follow-up study [file sj-docx-1-tan-10.1177_17562864211055694.docx]

**Table 3:** Univariate and multivariate analysis of different parameters of all spinal cord ependymomas as possible predictors for worse neurological outcome.

| **Parameters** | **Preoperative MCS (>2)** | **Age** | **Sex** | **WHO Grade (II/III)** | **Tumor volume (cm^3^)** | | **Spine segment (C/Th)** | **Number of vertebras (>2)** | **Pain (No)** | **Sensory disorder (Yes)** | **Paresis (Yes)** | **Ataxia (Yes)** | **Subtotal tumor resection** | **Symptom duration** |
| --- | --- | --- | --- | --- | --- | --- | --- | --- | --- | --- | --- | --- | --- | --- |
| ***Univariate analysis*** | ***P-value*** | ***P-value*** | ***P-value*** | ***P-value*** | ***Medians*** | ***P-value*** | ***P-value*** | ***P-value*** | ***P-value*** | ***P-value*** | ***P-value*** | ***P-value*** | ***P-value*** | ***P-value*** |
| **Postoperative MCS (score >2)** | **0.0005** | 0.892 | 0.483 | 0.115 | **3.0 vs 1.6** | **0.023** | **0.0005** | **0.0005** | **0.001** | 0.360 | **0.002** | **0.014** | 0.188 | 0.568* |
| **MCS at 3 months of follow-up (score >2)** | **0.0005** | 0.662 | 0.711 | ***0.098*** | **3.0 vs 1.6** | **0.026** | **0.0005** | **0.0005** | **0.0005** | 0.460 | **0.017** | **0.001** | **0.050** | 0.508** |
| **MCS at 6 months of follow-up (score >2)** | **0.0005** | 0.912 | 0.576 | 0.188 | **3.0 vs 1.6** | **0.024** | **0.0005** | **0.0005** | **0.006** | 0.614 | **0.006** | **0.0005** | **0.029** | 0.671*** |
| **MCS at 12 months of follow-up (score >2)** | **0.0005** | 0.513 | 0.424 | 0.423 | **4.4 vs 1.7** | **0.037** | **0.0005** | **0.0005** | **0.006** | 0.593 | **0.022** | **0.013** | **0.008** | 0.146# |
| **MCS at 24 months of follow-up (score >2)** | **0.0005** | 0.572 | 0.543 | 0.421 | **4.4 vs 1.7** | **0.041** | **0.0005** | **0.0005** | **0.006** | 0.439 | **0.016** | **0.009** | **0.010** | 0.233## |
| **MCS at 36 months of follow-up (score <2)** | **0.0005** | 0.536 | 0.279 | 0.277 | ***4.5 vs 1.9*** | ***0.070*** | **0.0005** | **0.001** | **0.004** | 0.394 | **0.009** | **0.004** | **0.002** | 0.414### |
| **Predictors** | **Preoperative MCS (>2)** | **Age** | **Sex** | **WHO Grade (II/III)** | **Tumor volume (cm^3^)** | | **Spine segment (C/Th)** | **Number of vertebras (>2)** | **Pain (No)** | **Sensory disorder (Yes)** | **Paresis (Yes)** | **Ataxia (Yes)** | **Subtotal tumor resection** | **Symptom duration** |
| ***Multivariate analysis*** | ***P-value*** | ***P-value*** | ***P-value*** | ***P-value*** | ***P-value*** | | ***P-value*** | ***P-value*** | ***P-value*** | ***P-value*** | ***P-value*** | ***P-value*** | ***P-value*** | ***P-value*** |
| **Postoperative MCS (score >2)** | **0.005** | -- | -- | -- | 0.248 | | **0.0005** | **0.011** | 0.453 | -- | **0.037** | 0.366 | **--** | -- |
| **MCS at 3 months of follow-up (score >2)** | **0.003** | -- | -- | 0.558 | 0.497 | | **0.013** | **0.006** | 0.303 | -- | 0.153 | **0.045** | 0.224 | -- |
| **MCS at 6 months of follow-up (score >2)** | **0.0005** | -- | -- | -- | 0.576 | | ***0.069*** | **0.0005** | **0.009** | -- | 0.217 | ***0.088*** | 0.206 | -- |
| **MCS at 12 months of follow-up (score >2)** | **0.0005** | -- | -- | -- | 0.409 | | **0.050** | **0.0005** | 0.133 | -- | ***0.081*** | 0.222 | ***0.058*** | -- |
| **MCS at 24 months of follow-up (score >2)** | **0.0005** | -- | -- | -- | 0.375 | | ***0.062*** | **0.01** | 0.359 | -- | **0.042** | 0.172 | ***0.052*** | -- |
| **MCS at 36 months of follow-up (score <2)** | **0.003** | -- | -- | -- | ***0.096*** | | **0.041** | 0.192 | 0.359 | -- | **0.019** | **0.025** | **0.003** | -- |
| ***: N=104; **: N=100; ***:N=99; #:N=94; ##:N=91; ###: N=81** | | | | | | | | | | | | | | |

Abbreviations: MCS= McCormick Scale; C= cervical; TH: thoracic

**Table 4:** Univariate and multivariate analysis of different parameters of spinal cord ependymomas of the cervical and thoracic spine as possible predictors for worse neurological outcome.

| **Parameters** | **Preoperative MCS (>2)** | **Age** | **Sex** | **WHO Grade (II/III)** | **Tumor Volume (cm^3^)** | | **Spine segment (cervical)** | **Number of vertebras (>2)** | **Pain (No)** | **Sensitive disorder (Yes)** | **Paresis (Yes)** | **Ataxia (Yes)** | **Subtotal tumor resection** |
| --- | --- | --- | --- | --- | --- | --- | --- | --- | --- | --- | --- | --- | --- |
| ***Univariate analysis*** | ***P-value*** | ***P-value*** | ***P-value*** | ***P-value*** | ***Medians*** | ***P-value*** | ***P-value*** | ***P-value*** | ***P-value*** | ***P-value*** | ***P-value*** | ***P-value*** | ***P-value*** |
| **Postoperative MCS (score >2)** | **0.015** | 0.585 | 0.488 | 0.338 | 2.8 vs 1.3 | ***0.051*** | 0.122 | **0.027** | 0.122 | *0.062* | *0.078* | 0.367 | 1.000 |
| **MCS at 3 months of follow-up (score >2)** | **0.026** | 0.417 | 0.638 | 0.322 | 2.9 vs 1.6 | ***0.087*** | 0.365 | **0.012** | 0.173 | 0.104 | 0.361 | 0.208 | 0.555 |
| **MCS at 6 months of follow-up (score >2)** | **0.013** | 0.699 | 0.643 | 0.213 | 2.9 vs 1.6 | ***0.087*** | 0.645 | **0.005** | ***0.064*** | 0.176 | 0.121 | 0.055 | 0.377 |
| **MCS at 12 months of follow-up (score >2)** | **0.038** | 0.863 | 1.000 | 0.302 | 4.4 vs 1.6 | ***0.092*** | 1.000 | **0.008** | 0.475 | 0.255 | 0.313 | 0.738 | 0.129 |
| **MCS at 24 months of follow-up (score >2)** | **0.034** | 0.759 | 1.000 | 0.311 | 4.4 vs 1.6 | ***0.100*** | 1.000 | **0.008** | 0.338 | 0.165 | 0.172 | 0.496 | 0.137 |
| **MCS at 36 months of follow-up (score >2)** | **0.047** | 0.797 | 0.435 | 0.569 | 4.5 vs 2.0 | 0.129 | 1.000 | **0.075** | 0.302 | 0.218 | ***0.069*** | 0.277 | ***0.014*** |
| **Predictors** | **Preoperative MCS (>2)** | **Age** | **Sex** | **WHO Grade (II/III)** | **Tumor Volume (cm^3^)** | | **Spine segment (cervical)** | **Number of vertebras (>2)** | **Pain (No)** | **Sensitive disorder (Yes)** | **Paresis (Yes)** | **Ataxia (Yes)** | **Subtotal tumor resection** |
| ***Multivariate analysis*** | ***P-value*** | ***P-value*** | ***P-value*** | ***P-value*** | ***P-value*** | | ***P-value*** | ***P-value*** | ***P-value*** | ***P-value*** | ***P-value*** | ***P-value*** | ***P-value*** |
| **Postoperative MCS (score >2)** | ***0.062*** | -- | -- | -- | 0.852 | | -- | **0.015,**  **aOR:3.3** | -- | 0.103 | ***0.054*** | -- | -- |
| **MCS at 3 months of follow-up (score >2)** | ***0.084*** | -- | -- | -- | 0.777 | | -- | **0.013,**  **aOR: 3.4** | -- | -- | -- | -- | -- |
| **MCS at 6 months of follow-up (score >2)** | ***0.073*** | -- | -- | -- | 0.790 | | -- | **0.004,**  **aOR:4.6** | **0.041,**  **aOR: 0.341** | -- | -- |  | -- |
| **MCS at 12 months of follow-up (score >2)** | 0.147 | -- | -- | -- | 0.700 | | -- | **0.005,**  **aOR: 4.5** | -- | -- | -- | -- | -- |
| **MCS at 24 months of follow-up (score >2)** | 0.125 | -- | -- | -- | 0.714 | | -- | **0.009,**  **aOR:4.2** | -- | -- | -- | -- | -- |
| **MCS at 36 months of follow-up (score >2)** | **0.022, aOR:4.4** | -- | -- | -- | -- | | -- | ***0.063*** | -- | -- | **0.036, aOR: 5.8** | -- | **0.06,**  **aOR: 9.3** |

**Table 5:** Univariate and multivariate analysis of different parameters of spinal cord ependymomas of the lumbosacral spine as possible predictors for worse neurological outcome.

| **Parameters** | **Preoperative MCS (>2)** | **Age** | **Sex** | **WHO Grade (II/III)** | **Tumor volume (cm^3^)** | | **Number of vertebras (>2)** | **Pain (No)** | **Sensitive disorder (Yes)** | **Paresis (Yes)** | **Ataxia (Yes)** | **Subtotal tumor resection** | **Intramedullary (Yes)** |
| --- | --- | --- | --- | --- | --- | --- | --- | --- | --- | --- | --- | --- | --- |
| ***Univariate analysis*** | ***P-value*** | ***P-value*** | ***P-value*** | ***P-value*** | ***Medians*** | ***P-value*** | ***P-value*** | ***P-value*** | ***P-value*** | ***P-value*** | ***P-value*** | ***P-value*** | ***P-value*** |
| **Postoperative MCS (score >2)** | **0.0005** | 0.665 | 1.000 | 1.000 | 6.9 vs 1.7 | 0.150 | ***0.079*** | 0.241 | 0.659 | 0.208 | 0.143 | 0.326 | 1.000 |
| **MCS at 3 months of follow-up (score >2)** | **0.0005** | 0.896 | 0.648 | 0.313 | 4.8 vs 1.6 | 0.141 | ***0.063*** | ***0.051*** | 1.000 | 0.217 | **0.005** | 0.280 | 1.000 |
| **MCS at 6 months of follow-up (score >2)** | **0.0005** | 0.896 | 0.648 | 0.313 | 4.8 vs 1.6 | 0.141 | ***0.063*** | ***0.051*** | 1.000 | 0.217 | **0.005** | 0.280 | 1.000 |
| **MCS at 12 months of follow-up (score >2)** | **0.0005** | 0.274 | 1.000 | 0.591 | 3.5 vs 1.7 | 0.348 | 0.346 | **0.028** | 1.000 | 0.187 | **0.003** | 0.243 | 0.394 |
| **MCS at 24 months of follow-up (score >2)** | **0.0005** | 0.297 | 1.00 | 0.598 | 3.5 vs 1.7 | 0.355 | 0.357 | **0.030** | 1.000 | 0.193 | **0.004** | 0.251 | 0.405 |
| **MCS at 36 months of follow-up (score >2)** | **0.0005** | 0.304 | 0.648 | 0.584 | 3.5 vs 1.8 | 0.416 | 0.411 | **0.029** | 1.000 | 0.226 | **0.005** | 0.291 | 1.000 |
| **Predictors** | **Preoperative MCS (>2)** | **Age** | **Sex** | **WHO Grade (II/III)** | **Tumor volume (cm^3^)** | | **Number of vertebras (>2)** | **Pain (No)** | **Sensitive disorder (Yes)** | **Paresis (Yes)** | **Ataxia (Yes)** | **Subtotal tumor resection** | **Intramedullary (Yes)** |
| ***Multivariate analysis*** | ***P-value*** | ***P-value*** | ***P-value*** | ***P-value*** | ***P-value*** | | ***P-value*** | ***P-value*** | ***P-value*** | ***P-value*** | ***P-value*** | ***P-value*** | ***P-value*** |
| **Postoperative MCS (score >2)** | **0.0005,**  **aOR:122** | -- | -- | -- | -- | | 0.335 | -- | -- | -- | -- | -- | -- |
| **MCS at 3 months of follow-up (score >2)** | 0.997 | -- | -- | -- | -- | | 0.997 | 0.593 | -- | -- | 0.248 | -- | -- |
| **MCS at 6 months of follow-up (score >2)** | 0.997 | -- | -- | -- | -- | | 0.997 | 0.593 | -- | -- | 0.248 | -- | -- |
| **MCS at 12 months of follow-up (score >2)** | -- | -- | -- | -- | -- | | -- | -- | -- | -- | -- | -- | -- |
| **MCS at 24 months of follow-up (score >2)** | -- | -- | -- | -- | -- | | -- | -- | -- | -- | -- | -- | -- |
| **MCS at 36 months of follow-up (score >2)** | -- | -- | -- | -- | -- | | -- | -- | -- | -- | -- | -- | -- |
